# Association of oral microbiota and digestive system cancers revealed by bidirectional two sample Mendelian randomization

**DOI:** 10.1007/s12672-026-04387-5

**Published:** 2026-01-06

**Authors:** Ya-Jun Zhang, Qian-Yu Tian, Cai-E. Wang, Shu-Xun Wei

**Affiliations:** 1 Department of Pharmacy, The First Affiliated Hospital, and College of Clinical Medicine of Henan University of Science and Technology, 24 Jinghua Road, Jianxi District, Luoyang, 471003 China; 2 Department of Vascular Surgery, Eastern Hepatobiliary Surgery Hospital, Naval Medical University/Second Military Medical University, 225 Changhai Road, Yangpu District, Shanghai, 200438 China

**Keywords:** Causality, Digestive system cancers, Oral microbiota, Mendelian randomization, SNPs

## Abstract

**Background:**

Growing evidence suggests that there is a link between the oral microbiota and the development of digestive system cancers (DSCs). Nonetheless, the causal relationship between the oral microbiota and DSCs has yet to be established.

**Methods:**

To evaluate the causal relationship between oral microbiota and DSCs, we employed Genome-wide association study (GWAS) summary statistics for both oral microbiota and DSCs, in conjunction with bidirectional two-sample Mendelian randomization (MR) analysis. Single nucleotide polymorphisms, which were free from confounding factors, were used as instrumental variables to infer causation. Sensitivity analyses were conducted to assess the robustness of our findings. This study employed datasets encompassing a wide range of cancer cases and controls, with an emphasis on Asian populations.

**Results:**

Our analysis of 116 oral microbiota (65 from tongue dorsum and 51 from saliva) uncovered intricate causal associations with seven types of DSCs. We discovered that the genus *TM7x* (OR > 1, adjusted *p* < 0.05) poses a risk for hepatic bile duct cancer, and the genus *Leptotrichia* (OR = 6.38, 95%CI = 1.84–22.10, adjusted *p* < 0.05) poses a risk for pancreatic cancer. Furthermore, reverse MR analysis showed that DSCs influence the relative abundance of certain oral microbiota strains.

**Conclusion:**

Our MR analysis has confirmed that there is a causal association between the oral microbiota and DSCs. This finding offers potential for creating novel microbial markers and treatments that modify the microbiota specifically for DSC patients.

**Supplementary Information:**

The online version contains supplementary material available at 10.1007/s12672-026-04387-5.

## Introduction

Worldwide, cancer was responsible for 18% of all deaths, making it the second leading cause of mortality following heart diseases [1]. Among these, colorectal cancer (CRC), gastric cancer (GC), hepatic cancer, esophageal cancer and pancreatic cancer (PC) are the most commonly diagnosed digestive system cancers (DSCs), with both the incidence and mortality rates rising worldwide. Despite significant advancements in the early screening of DSCs recently, the survival rates remain low [2].

The oral cavity hosts over 700 bacterial species and is the gateway to the digestive tract, creating a direct microbial link. This connection allows oral bacteria and their metabolites to continuously seed downstream organs, including the esophagus, stomach, liver, and pancreas. Evidence suggests these translocated microbes can promote carcinogenesis by modulating local immunity, causing chronic inflammation, and producing genotoxins [5, 6]. Observational studies have linked specific oral taxa to diseases like PC [7, 8]. However, these findings are often limited to single cancer types and are susceptible to confounding and reverse causality. Therefore, a systematic investigation across all DSCs is needed to clarify the role of the oral microbiota in carcinogenesis and to inform new prevention and diagnostic strategies.

Mendelian randomization (MR) offers a way to assess causality while overcoming the limitations of observational studies. This approach uses randomly allocated germline genetic variants as unconfounded proxies for microbial exposures [9]. By mimicking a randomized trial, MR is well-suited to this task. First, genetic instruments are independent of lifestyle and environmental confounders that typically bias observational findings. Second, because they are fixed at birth, they prevent reverse causality, where the disease itself might alter the microbiota [10]. Here, we apply a two-sample MR design to large GWAS datasets to estimate the causal effects of the oral microbiota on DSC risk. This work moves beyond simple association to test for causal inference.

## Method

### Study design

**Fig. 1:**
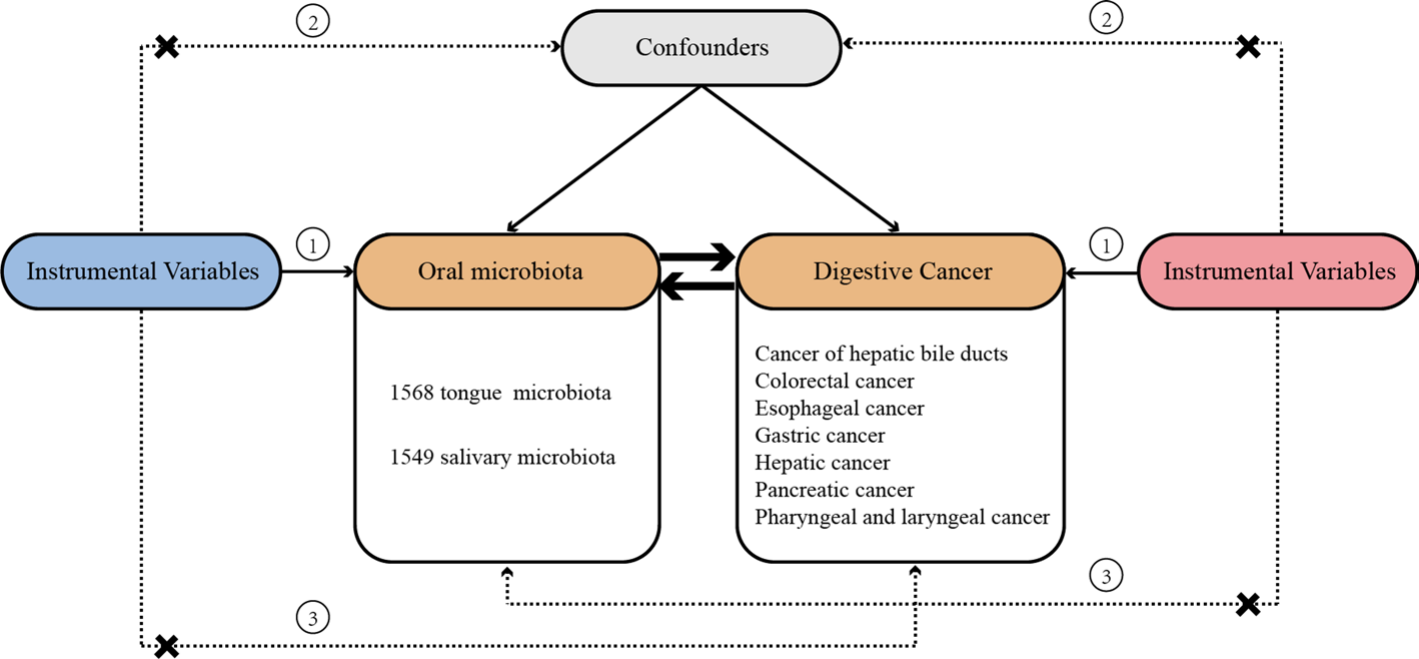
The overview of the research workflow

In the context of this study, the two-sample MR method was employed to evaluate the possible causal link between oral microbiota and DSCs. Single nucleotide polymorphisms (SNPs) were utilized as instrumental variables (IVs). To ensure the rigor of a two-sample MR analysis, it must satisfy three key assumptions: (1) The selected IVs are strongly associated with the oral microbiota; (2) The selected IVs are not related to confounders; (3) The selected IVs influence DSCs solely through their effect on the oral microbiota. To ascertain the direction of the causal relationship between oral microbiota and DSCs, bidirectional MR and the Steiger test were utilized. Figure 1 provides a comprehensive overview of the methodology.

### Data sources and study population

The dataset of the oral microbiota that we used marks the first analysis of the oral microbiota in East Asian populations. It comprises of 2017 samples from the tongue dorsum and 1915 saliva samples, and it covers a total of 10,098,668 loci [11]. Data for 1568 sets of tongue dorsum microbiota data and 1549 sets of saliva microbiota data were obtained from the China National GeneBank DataBase (CNGBdb, CNP0001664, https://db.cngb.org/) for this analysis. These datasets were chosen based on strict selection criteria including: (1) a variant calling rate of at least 98%; (2) a mean sequencing depth exceeding 20×; (3) the absence of population stratification in principle component analysis (PCA); (4) the exclusion of related individuals via pairwise identity by descent estimates; (5) a minor allele frequency (MAF) of at least 0.01. For an in-depth look at the methods used in this study, including sample collection, sequencing techniques, microbiota trait creation, and observational and genotyping studies, please see the work of Liu et al. [11].

For reverse analysis, the GWAS data of seven types of DSCs (cancer of hepatic bile ducts, CRC, esophageal cancer, GC, hepatic cancer, PC, and pharyngeal and laryngeal cancer) that we utilized were analyzed by Sakaue et al. [12]. All GWAS data were downloaded from the BBJ PheWeb (https://pheweb.jp/) website [13]. In their study, Sakaue et al. analyzed 178,726 participants of East Asian descent, with the sample selection criteria being estimated based on PCA. To further refine the genotypic data, the Minimac3 software was employed, leveraging Phase 3 Version 5 genotype data from the 1000 Genomes Project (*n* = 2,504) and whole-genome sequencing data from Japan (*n* = 1,037), incorporating a total of 13,530,797 variants for analysis [14]. For binary traits, the analysis and correction for data imbalances were conducted using a generalized linear mixed model in SAIGE (v.0.37), including age, age^2, sex, age×sex, age^2×sex, and the top 20 principal components as covariates [15]. Table 1 provides the specifics of the GWAS datasets that were chosen for this study. To further draw reliable conclusions, we utilized PhenoScanner (http://www.phenoscanner.medschl.cam.ac.uk/) to search for confounding SNPs of all.

**Table 1:** The GWAS datasets for DSCs

Trait	Class	Sample size	Case	Control	Population	Data source
Oral microbiota	Tongue dorsum	2017	–	–	East Asian	34,873,157
	Saliva	1915	–	–	East Asian	34,873,157
DSCs	Cancer of hepatic bile ducts	159,619	418	159,201	East Asian	34,594,039
	Colorectal cancer	167,691	8,305	159,386	East Asian	34,594,039
	Esophageal cancer	160,589	1,388	159,201	East Asian	34,594,039
	Gastric cancer	167,122	7,921	159,201	East Asian	34,594,039
	Hepatic cancer	161,323	2,122	159,201	East Asian	34,594,039
	Pancreatic cancer	159,700	499	159,201	East Asian	34,594,039
	Pharyngeal and laryngeal cancer	178,726	300	178,426	East Asian	34,594,039
UKBPPP	2940 proteins	262	–	–	East Asian	37,794,186
Xu et al.	1602 proteins and peptides	2410	–	–	East Asian	36,797,296

*DSCs* digestive system cancers, *UKBPPP* UK Biobank Pharma Proteome Project

tumor risk factors and excluded them [16]. The selection of potential confounders was based on a review of established risk factors for the digestive system cancers under study. These included smoking, alcohol use, obesity, and inflammatory bowel disease (IBD), among others [17–21]. SNPs found to be associated with any of these risk factors were excluded from the analysis. The detailed results of this screening, including the number of SNPs excluded for each microbial taxon, are provided in Supplementary Table 1.

### Instrumental variable selection in MR analysis

To ensure accuracy in inferring the causal relationship between oral microbiota and DSCs, we applied the following criteria to select IVs: (1) IVs were selected based on their strong association with exposure, requiring a p-value < 5 × 10^− 8^; (2) SNPs were excluded to mitigate linkage disequilibrium, using thresholds of r2 = 0.001 and kb = 10,000; (3) SNPs associated with confounding variables were identified and excluded via the PhenoScanner website; (4) SNPs were chosen with a MAF greater than 0.01; (5) SNPs were also required to have an F-statistic greater than 10 [22]. The F-statistic is calculated using the formula, where N represents the number of samples in GWAS research, and R2 represents the degree of exposure explained by IVs [23]:

$$\:\mathrm{F}=(\mathrm{R}2/(1-\mathrm{R}2\left)\right)\mathrm{*}\left(\right(\mathrm{s}\mathrm{a}\mathrm{m}\mathrm{p}\mathrm{l}\mathrm{e}\mathrm{s}\mathrm{i}\mathrm{z}\mathrm{e}.\mathrm{e}\mathrm{x}\mathrm{p}\mathrm{o}\mathrm{s}\mathrm{u}\mathrm{r}\mathrm{e}-\mathrm{k}-1)/\mathrm{k}))$$

### Bidirectional two-sample MR analysis

To investigate causal relationships, we employed a bidirectional two-sample MR design. Our primary analysis used the inverse variance weighted (IVW) method, which provides the most precise estimate assuming no unbalanced horizontal pleiotropy. We supplemented this with four sensitivity analyses—MR-Egger, weighted median, simple mode, and weighted mode—to assess the robustness of our findings [24]. Discrepancies between the IVW and sensitivity analyses would indicate potential pleiotropy, requiring a more cautious interpretation of the results. For exposures with a single instrumental SNP, we used the Wald Ratio method. To control for multiple testing, we applied the Benjamini-Hochberg False Discovery Rate (FDR) procedure to the p-values for each cancer type separately [25]. An association was considered significant if the FDR-adjusted p-value (q-value) was < 0.05. This method was chosen over the more stringent Bonferroni correction to better accommodate the correlated nature of microbiome data and retain statistical power. Both raw and adjusted p-values are provided in Supplementary Table 2. Additionally, for exposures involving multiple SNPs, we assessed heterogeneity, pleiotropy, and conducted leave-one-out analyses using the TwoSampleMR package [26, 27].

### Mediation analysis

To further explore whether the oral microbiota influences gastrointestinal tumors through other mediators, we conducted a mediation analysis, identifying proteins from Asian populations as potential intermediaries. This analysis was based on data from the UK Biobank Pharma Proteome Project (UKBPPP) and insights from the article “Genome-wide genotype-serum proteome mapping provides insights into the cross-ancestry differences in cardiometabolic disease susceptibility” [28, 29]. The selection criteria for protein instrumental variables (PIVs) mirrored those for oral microbiota IVs, with an FDR correction being applied to the MR results for each type of tumor.

### Statistical analysis

Statistical analyses were performed utilizing R software (version 4.2.2). The packages employed for data cleaning, MR analysis, and plotting include “plinkbinr”, “rlist”, “TwoSampleMR”, and “ggplot2”, among others [30]. Unless otherwise specified within the text, all statistically significant thresholds are established at a p-value of < 0.05.

## Result

### SNP selection

Based on the stringent criterion of *p* < 5 × 10^− 8^, we preliminarily selected IVs for oral microbiota and then utilized the “TwoSampleMR” package to address linkage disequilibrium. Additionally, in our effort to identify robust IVs and minimize bias, we chose SNPs with an F-value exceeding 10.

The SNPs incorporated in this research are representative of species. Considering that certain bacterial traits lacked specific species names, our analysis was accordingly conducted at the genus or family level that aligns with the selected bacteria. To further mitigate confounding factors, we reviewed risk factors for each tumor type as mentioned in review articles, utilizing PhenoScanner to identify and subsequently exclude SNPs associated with these factors from our analysis. Consequently, through these meticulous selection criteria, we identified 68 host-derived SNPs from the 65 tongue dorsum microbiota samples and 52 host-derived SNPs from the 51 saliva microbiota samples. We used the same filtering method to select 29 SNPs as IVs for reverse MR from 4 types of DSCs.

### The bidirectional causal associations between oral microbiota and DSCs

Bidirectional MR analysis was conducted to examine the causal relationships between oral microbiota and seven distinct types of DSCs. Specifically, we focused on the results of the MR-Egger method as well as the IVW method. We used the Steiger test to confirm the assumed causal direction from oral microbiota to DSCs. For all significant associations, the genetic instruments explained substantially more variance in the exposure than in the outcome. This finding, combined with significant Steiger p-values, strongly supports the proposed causal pathway. Detailed results are available in Supplementary Table 3. To provide a comprehensive overview, all significant findings are detailed in Supplementary Tables 4 and Supplementary Table 5.

### Pharyngeal and laryngeal cancer

**Fig. 2:**
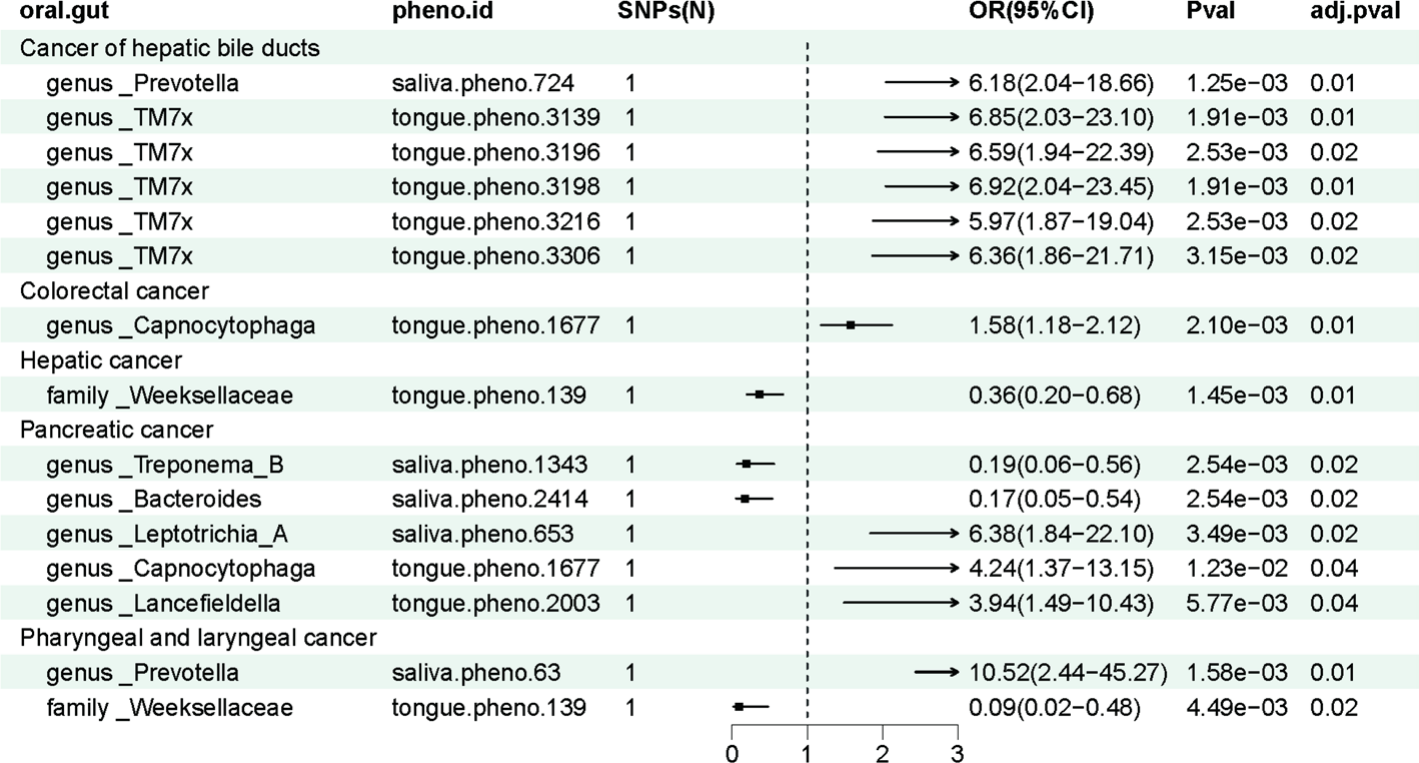
The significant causal effect of oral microbiota on DSCs calculated using the MR-Egger method. DSCs: digestive system cancers; SNPs (n): number of single nucleotide polymorphisms

We discovered that the genetically predicted relative abundance of genus *Prevotella* (OR = 10.52, 95%CI = 2.44–45.27, adjusted *p* < 0.05) showed a positive association with the risk of pharyngeal and laryngeal cancer within the set of IVs (*p* < 5 × 10^− 8^). The genetically predicted relative abundance of family *Weeksellaceae* (OR = 0.09, 95%CI = 0.02–0.48, adjusted *p* < 0.05) demonstrated a negative association with the risk of pharyngeal and laryngeal cancer (Fig. 2).

A Based on the findings from the reverse MR analysis, we did not find any genetic predisposition to pharyngeal and laryngeal cancer that was causally associated with oral microbiota.

### Esophageal cancer

Within the set of IVs (*p* < 5 × 10^− 8^), no genetic predisposition to oral microbiota was identified as causally linked to esophageal cancer.

**Fig. 3:**
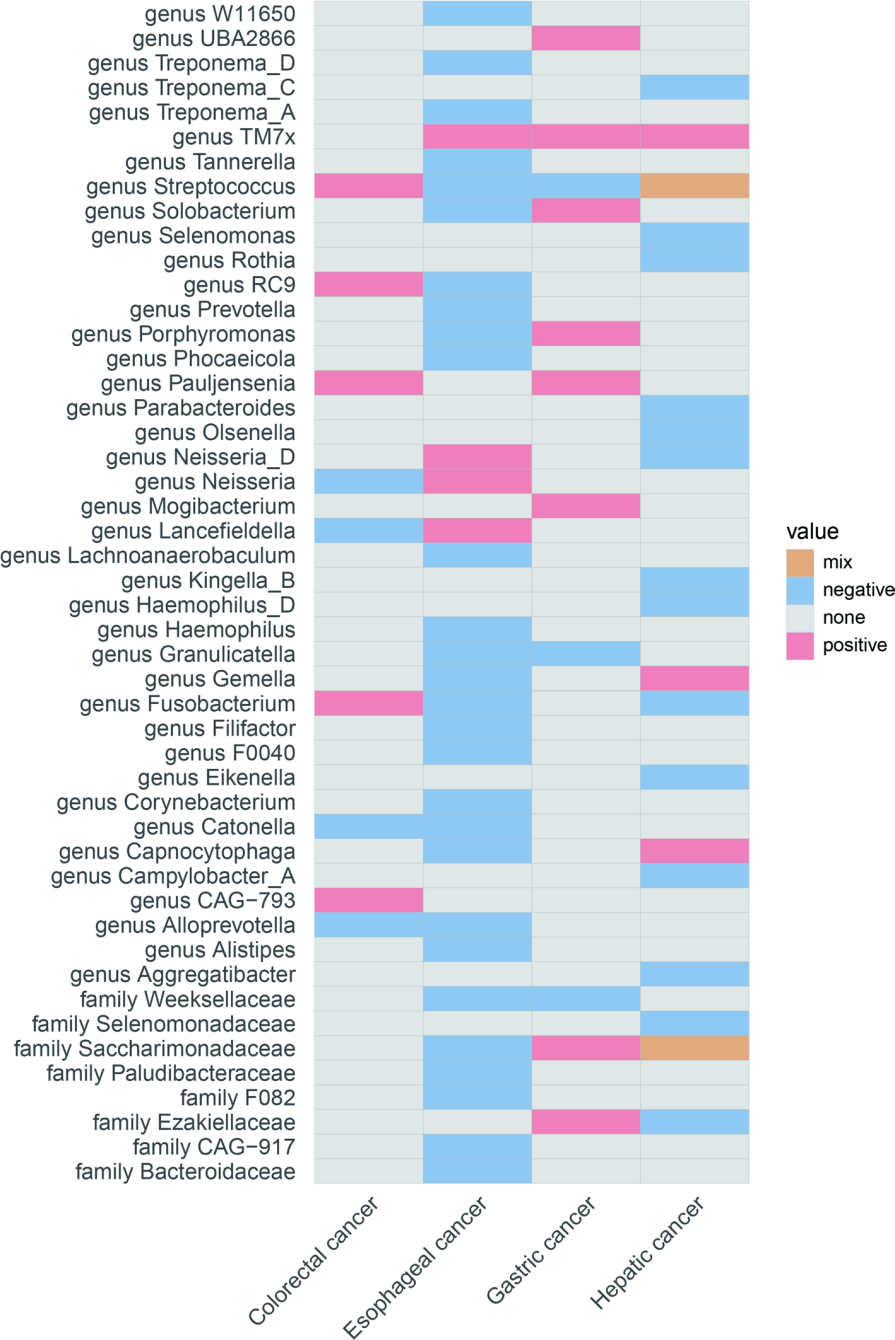
The significant causal effect of DSCs on oral microbiota calculated using the IVW method. Results categorized by species ORs within each genus or family: mix (ORs > 1 and < 1), positive (all ORs > 1), negative (all ORs < 1), none (no OR values). Detailed data in Supplementary Table 5. DSCs: digestive system cancers

According to the results of reverse MR analysis, the genetic predisposition to esophageal cancer was found to be positively associated with the relative abundance of the genera *Lancefieldella*, *Neisseria*, *Neisseria_D*, and *TM7x* (OR > 1, adjusted *p* < 0.05). Conversely, the genetic predisposition to esophageal cancer was negatively associated with the relative abundance of several bacterial genera (*Streptococcus*,* Granulicatella*,* Prevotella*,* F0040*,* Phocaeicola*,* Alloprevotella*,* Solobacterium*,* Filifactor*,* Capnocytophaga*,* Fusobacterium*,* Gemella*,* Catonella*,* Lachnoanaerobaculum*,* Corynebacterium*,* Haemophilus*,* Porphyromonas*,* Alistipes*,* Tannerella*,* Treponema_A*,* Treponema_D*,* RC9*,* W11650*) and families (*Bacteroidaceae*,* CAG-917*,* F082*,* Paludibacteraceae*,* Saccharimonadaceae*,* Weeksellaceae*) (OR < 1, adjusted *p* < 0.05) (Fig. 3).

### Gastric cancer

Within the set of IVs (*p* < 5 × 10^− 8^), no genetic predisposition to oral microbiota was identified as causally linked to GC.

According to the results of reverse MR analysis, the genetic susceptibility to GC was discovered to be positively correlated with the relative abundance of the genera *Pauljensenia*, *Mogibacterium*, *Solobacterium*, *Porphyromonas*, *TM7x*, and *UBA2866*,as well as the families *Ezakiellaceae* and *Saccharimonadaceae* (OR > 1, adjusted *p* < 0.05). Conversely, the genetic predisposition to GC was negatively associated with the relative abundance of genus *Granulicatella*, genus *Streptococcus* and family *Weeksellaceae* (OR < 1, adjusted *p* < 0.05) (Fig. 3).

### Colorectal cancer

Within the set of IVs (*p* < 5 × 10^− 8^), our findings indicated that the genetically predicted relative abundance of genus *Capnocytophaga* (OR = 1.58, 95%CI = 1.18–2.12, adjusted *p* < 0.05) was positively associated with the risk of CRC (Fig. 3).

Reverse MR analysis results revealed a positive association between genetic susceptibility to CRC and the relative abundance of the genera *Pauljensenia*,* CAG-793*,* Fusobacterium*,* Streptococcus*, and *RC9* (OR > 1, adjusted *p* < 0.05). In contrast, genetic susceptibility to CRC showed a negative correlation with the relative abundance of the genera *Lancefieldella*,* Alloprevotella*,* Catonella*, and *Neisseria* (OR < 1, adjusted *p* < 0.05) (Fig. 3).

### Hepatic cancer

Within the set of IVs (*p* < 5 × 10^− 8^), our analysis revealed a negative association between the genetically estimated relative abundance of the family *Weeksellaceae* (OR = 0.36, 95%CI = 0.20–0.68, adjusted *p* < 0.05) and the risk of hepatic cancer (Fig. 3).

Reverse MR analysis indicated that a genetic predisposition to hepatic cancer is positively correlated with the relative abundance of the genera *Capnocytophaga*, *Gemella* and *TM7x* (OR > 1, adjusted *p* < 0.05). Conversely, the genetic predisposition to hepatic cancer was negatively associated with the relative abundance of the family *Ezakiellaceae* and the genera *Olsenella*, *Campylobacter_A*, *Fusobacterium*, *Rothia*, *Kingella_B*, *Eikenella*, *Neisseria_D*, *Haemophilus_D*, *Aggregatibacter*, *Selenomonas*, *Parabacteroides*, and *Treponema_C* (OR < 1, adjusted *p* < 0.05) (Fig. 3).

### Cancer of hepatic bile ducts

Within the set of IVs (*p* < 5 × 10^− 8^), our findings revealed that the genetically predicted relative abundance of genus *TM7x* (OR > 1, adjusted *p* < 0.05) and genus *Prevotella* (OR = 6.18, 95%CI = 2.04–18.66, adjusted *p* < 0.05) were positively associated with the risk of cancer of hepatic bile ducts (Fig. 3).

According to the results of reverse MR analysis, we did not find any genetic predisposition to cancer of hepatic bile ducts that was causally associated with oral microbiota.

### Pancreatic cancer

Among the set of IVs (*p* < 5 × 10^− 8^), our analysis identified that the genetically predicted relative abundance of the genera *Leptotrichia_A* (OR = 6.38, 95%CI = 1.84–22.10, adjusted *p* < 0.05), *Capnocytophaga* (OR = 4.24, 95%CI = 1.37–13.15, adjusted *p* < 0.05) and *Lancefieldella* (OR = 3.94, 95%CI = 1.49–10.43, adjusted *p* < 0.05) were positively associated with the risk of PC. Conversely, the genetically predicted relative abundance of genus *Treponema_B* (OR = 0.19, 95%CI = 0.06–0.56, adjusted *p* < 0.05) and genus *Bacteroides* (OR = 0.17, 95%CI = 0.05–0.54, adjusted *p* < 0.05) was negatively associated with the risk of PC (Fig. 3).

Based on the outcomes of the reverse MR analysis, no genetic predisposition to PC was found to be causally linked to oral microbiota.

### Exploring the mediating effect of serum proteins between oral microbiota and DSCs

Considering the possible contribution of serum proteome on the progress from the oral microbiota to DSCs, we analyzed serum proteins from the Asian population background as mediating factors. Despite this, the corrected positive results from MR analysis between the two serum protein data sets and DSCs did not intersect with the positive bacterial communities in the above results (Supplementary Tables 6and Supplementary Table 7). Thus, we preliminarily rule out the possibility that oral microbiota may affect the occurrence and development of DSCs through serum proteins.

### Sensitivity analyses

All pleiotropy tests yielded non-significant results (*p* > 0.05), which implies that horizontal pleiotropy is unlikely to be present in the IVs (Supplementary Table 8). To further substantiate this, we applied both the IVW method and MR-Egger regression to assess heterogeneity. Consistently, the outcomes of these tests were also non-significant, reinforcing the conclusion that the instrumental variables exhibit homogeneity (Supplementary Table 9).

## Discussion

To the best of our knowledge, this research is one of the pioneering attempts to systematically assess the causal relationships between the oral microbiota and DSCs through a bidirectional two-sample MR study. This methodology offered fairly robust evidence that the genetically predicted abundance of certain oral microbiota was associated with the risk of various DSCs. Additionally, the reverse MR analysis revealed notable associations between genetic susceptibility to DSCs and the relative abundance of various oral microbiota. These results imply that public health interventions targeting the reduction of cancer risk could be beneficial.

Current studies suggest several ways in which oral microbes may contribute to the emergence and progression of tumors. Firstly, they can initiate and intensify persistent inflammation, thus fostering tumor growth through their own molecular constituents [31, 32]. Secondly, these microbes can influence cellular growth and programmed cell death, achieving this by interfering with the normal progression of the cell cycle and altering pathways involved in tumor signaling [33–35]. Thirdly, oral microbes have the capability to metabolize various substances, including sulfides, nitrosamines, hydroxyl radicals, acetaldehyde, deoxycholic acid, and certain toxins. This metabolism results in by-products that may influence the initiation, spread, and recurrence of tumors [36–38]. Lastly, these microbes play a critical role in modulating the immune system’s response to the host, serving as a key factor in the body’s defense against tumors.

As illustrated by our MR analysis, we discovered that the genus *Prevotella* is a potential risk factor for cancers of the pharynx, larynx, and hepatic bile ducts. Conversely, the family *Weeksellaceae* appears to serve as a protective element against cancers affecting the liver and the pharyngeal and laryngeal regions. Additionally, our findings indicate the genus *Capnocytophaga* as a risk factor for both colorectal and pancreatic cancers. On the other hand, the genus *Treponema* and *Bacteroides* seem to offer a protective effect against PC, whereas the genus *Leptotrichia* and *Lancefieldella* were associated with an increased risk of the same. In summary, our findings support the hypothesis that different microbial species may have varying influences on cancer development, potentially through mechanisms involving the tumor microenvironment.

For the first time, our findings indicated that the genus *TM7x* is a risk factor for cancer of hepatic bile ducts. *TM7x* is distinguished from other bacterial species by its extraordinarily minuscule stature and its symbiotic existence, which is characterized by its adherence to the surface of its host bacterium. Characterized by a greatly diminished genome, *TM7x* is incapable of synthesizing its own amino acids, vitamins, or cell wall precursors, making it completely dependent on the parasitization of other oral bacteria for its essential nutrients [39]. As an epiparasite that is indispensable for its own survival yet harmful to its host, *TM7x* compromises the host bacterium’s wellbeing, eliciting stress responses and impeding its cellular growth and division. In extreme cases, such as nutrient depletion in a batch culture, *TM7x* can even induce host cell lysis [40, 41]. However, the extent to which *TM7x* may influence the progression of hepatic bile duct cancer remains to be elucidated through further experimental research.

Additionally, we found that the genus *Leptotrichia* is a risk factor for PC. To further explore the oral dysbiosis associated with PC in Chinese subjects, Wei et al. conducted an evaluation of the oral microbiota in patients with PC compared to healthy controls, using clinical presentation as a metric [42]. This investigation reaffirmed our findings; Wei et al. observed a correlation between *Leptotrichia* and an increased risk of PC [42]. Conversely, these results contrast with those presented by Fan et al., which suggested that the presence of *Leptotrichia* was linked to a reduced risk of PC (OR = 0.87, 95% CI 0.79–0.95) [43]. The discrepancy in findings likely stems from population differences; our study focused on East Asians, whereas the other included multiple ancestries. Furthermore, the study designs differ. Our MR analysis estimates lifetime causal effects, while observational studies identify associations prone to confounding and reverse causality. This suggests that the effects of specific oral microbiota may be context-dependent rather than universal.

Utilizing reverse MR analysis, we uncovered links between several specific oral bacterial genera and the incidence of esophageal cancer, GC, hepatic cancer, and CRC. Notably, our research revealed a significant association between the heightened presence of the genus *TM7x* in the oral cavity and the occurrence of esophageal cancer, GC, and hepatic cancer. Furthermore, our findings indicated a negative correlation between esophageal and hepatic cancers with the relative abundance of genus Fusobacterium, whereas CRC demonstrated a positive correlation. This concurs with previously published data indicating that high levels of genus *Fusobacterium* in CRC patients are implicated in tumor aggressiveness, including metastasis, recurrence, resistance to chemotherapy, and diminished radiotherapy effectiveness [44–47]. However, our findings also revealed contradictory associations for this genus in different cancer types, highlighting the highly context-dependent nature of microbial effects. The oncogenic potential of a microbe may not be an intrinsic property but rather depends on the local tissue microenvironment and tumor heterogeneity. Moreover, our study indicated that both esophageal cancer and GC were linked to a reduced presence of genus *Streptococcus*, contrasting with CRC, which showed a connection with increased levels of this genus. The *Streptococcus* genus is associated with mediating inflammation and creating an immunosuppressive tumor microenvironment (TME) that is characterized by a prevalence of myeloid-derived suppressor cells (MDSCs) and tumor-associated macrophages (TAMs), factors that contribute to the advancement of CRC [48, 49]. This research has pointed out an imbalance in the oral microbiota associated with DSCs, potentially offering valuable information and prospective diagnostic biomarkers for individuals diagnosed with DSCs. The observed reverse causality—the effect of cancer on the oral microbiota—is likely multifactorial. It may arise from the disease’s systemic effects (e.g., chronic inflammation) or, more directly, from its treatment. Chemotherapy and radiotherapy are potent disruptors of oral homeostasis. A critical confounder is the mixed composition of the GWAS cohorts, which include both treated and untreated patients, making it impossible to isolate the true effect of the disease from the iatrogenic effects of its therapy. The cancer GWAS cohorts likely include treated patients as they are typically not stratified by treatment status, introducing a significant risk of collider bias from the effects of therapy.

However, it is noteworthy that the absence of several experimentally validated oral microbes, such as the genus *Peptostreptococcus*, which are recognized for their involvement in dysregulated signaling pathways associated with CRC, raises significant questions about our findings [50]. This discrepancy may be attributed to the specific genetic variants chosen for our MR analysis, which might not correlate strongly with the abundance of *Peptostreptococcus* in the studied population. Weakly associated genetic instruments can lead to biased estimates, potentially overlooking significant microbial taxa that play critical roles in cancer development. Additionally, our reliance on summary-level data may limit the detection of certain taxa, particularly those present at lower abundances. The inherent complexity of microbial communities means that some important microbes may not be adequately represented in our analysis [51]. Furthermore, the roles of different microbes can vary significantly across cancer types and populations, influenced by environmental, immunological, and dietary factors [52–54].

Indeed, a single type of bacterium can play contrasting roles in cancer development by engaging different mechanisms that can either suppress or promote tumor growth. The connection between the oral microbiota and cancer is far-reaching with substantial public health and clinical implications. Monitoring changes in the oral microbiota could be integrated into regular cancer risk evaluations in the future. For patients exhibiting an imbalanced oral microbiota, early intervention tactics, including dietary modifications, use of prebiotics and probiotics, and potentially microbiota transplantation, might offer considerable benefits.

The lack of overlap between our identified oral taxa and mediating serum proteins suggests the true causal pathways were not captured by our analysis. This may be attributed to the microbiota acts through alternative biological mechanisms—such as producing pro-tumorigenic metabolites or modulating local immunity—that were not measured. Alternatively, it may reflect a fundamental methodological limitation: systemic protein levels are often a poor proxy for critical, tissue-specific molecular events within the local tumor microenvironment.

This research boasts several strengths. Firstly, it is the first study to investigate the causal relationship between the oral microbiota and DSCs. To confirm this connection, MR analysis was conducted, allowing us to investigate and provide genetic evidence for the causal relationship between oral microbiota and DSCs. This approach effectively eliminated the influence of confounding factors and reversed causation in causal inference. Moreover, FDR correction was employed to minimize the likelihood of false positives. Finally, our study conducted a detailed analysis at the level of species, which resulted in more accurate and reliable results.

The current study also has several limitations. Firstly, our study’s primary limitation is insufficient statistical power. Our stringent adherence to a genome-wide significance threshold (*p* < 5 × 10⁻⁸) to ensure instrument validity left many taxa with few or no eligible SNPs. Consequently, our analysis was likely underpowered to detect weak to moderate effects; therefore, our non-significant findings should be considered inconclusive rather than definitive evidence of no effect. This underscores the critical need for larger, more powerful GWAS of the oral microbiota to enable more comprehensive causal inference. Secondly, while we have sought to elucidate the causal relationship between oral microbiota and DSCs, this relationship may vary across different cancer pathological types. Due to our reliance on summary-level data, we could not perform subgroup analyses, indicating that further MR studies with individual-level data are essential to uncover more nuanced insights. Thirdly, while MR analysis can establish causality, it cannot elucidate the underlying biological mechanisms. Therefore, although our findings suggest a statistical link, in vitro and in vivo experiments are required to uncover the precise functional pathways through which these microbes influence carcinogenesis. Fourthly, our exposure data, derived from tongue and saliva GWAS, may not represent the entire oral microbiome, particularly distinct niches like subgingival plaque. Consequently, our findings reflect an aggregated causal signal and could mask important site-specific effects. Future GWAS focusing on specific oral sites are needed to dissect these more localized relationships. Lastly, since the study population was predominantly of Asian ancestry, the applicability of our findings to other ancestries requires careful consideration. To enhance the generalizability of these results, future MR studies should aim to include a more diverse cohort, encompassing both global population and each country populations.

## Conclusion

In conclusion, our study conducted a thorough assessment of the causal association between the oral microbiota and DSCs using bidirectional two-sample MR analysis. Our findings suggest a potential causal relationship between the oral microbiota and DSCs, opening avenues for the identification of novel microbial markers. For clinicians, this implies that modulating the oral microbiota could be a promising strategy for DSCs prevention, and alterations in the abundance of oral microorganisms might serve as potential biomarkers for tumorigenesis prediction. To fully understand this complex interaction, future research is necessary to elucidate the underlying mechanisms.

## Supplementary Information

Supplementary Material 1: Supplementary Table 1. Details of genetic instruments excluded due to association with potential confounders identified via PhenoScanner. Supplementary Table 2. Results of the Mendelian randomization analysis for the association between gut microbial taxa and DSCs, including raw and FDR-adjusted P-values. Supplementary Table 3. Assessment of instrument strength (F-statistics) for the genetic instruments used in the Mendelian randomization analysis. Supplementary Table 4. The significant causal effect of oral microbiota on DSCs calculated using the MR-Egger method. Supplementary Table 5. The significant causal effect of DSCs on oral microbiota calculated using the IVW method. Supplementary Table 6. The causal effect of UKBPPP pQTLs on oral microbiota. Supplementary Table 7. The causal effect of 36797296 pQTLs on oral microbiota. Supplementary Table 8. Results of horizontal pleiotropy test. Supplementary Table 9. Results of heterogeneity.

## Data Availability

Data is provided within the manuscript or supplementary information files.

## Abbreviations

DSCs: Digestive system cancers
GWAS: Genome-wide association study
MR: Mendelian randomization
CRC: Colorectal cancer
GC: Gastric cancer
PC: Pancreatic cancer
SNPs: Single nucleotide polymorphisms
IVs: Instrumental variables
PCA: Principle component analysis
MAF: Minor allele frequency
BBJ: BioBank Japan
UKBPPP: UK Biobank Pharma Proteome Project
PIVs: Protein instrumental variables
TME: Tumor microenvironment
MDSCs: Myeloidderived suppressor cells
TAMs: Tumorassociated macrophages
pQTLs: Protein quantitative trait loci
