# Supplementary material for: Association of oral microbiota and digestive system cancers revealed by bidirectional two sample Mendelian randomization
Source: Discov Oncol. 2026 Jan 6;17:223. doi: 10.1007/s12672-026-04387-5 (PMC12868531; doi:10.1007/s12672-026-04387-5)
Supplement: Supplementary file 1 — Supplementary Material 1: Supplementary Table 1. Details of genetic instruments excluded due to association with potential confounders identified via PhenoScanner. Supplementary Table 2. Results of the Mendelian randomization analysis for the association between gut microbial taxa and DSCs, including raw and FDR-adjusted P-values. Supplementary Table 3. Assessment of instrument strength (F-statistics) for the genetic instruments used in the Mendelian randomization analysis. Supplementary Table 4. The significant causal effect of oral microbiota on DSCs calculated using the MR-Egger method. Supplementary Table 5. The significant causal effect of DSCs on oral microbiota calculated using the IVW method. Supplementary Table 6. The causal effect of UKBPPP pQTLs on oral microbiota. Supplementary Table 7. The causal effect of 36797296 pQTLs on oral microbiota. Supplementary Table 8. Results of horizontal pleiotropy test. Supplementary Table 9. Results of heterogeneity. [file 12672_2026_4387_MOESM1_ESM.zip › STROBE-MR-checklist.pdf]

3.STROBE-MR checklist of recommended items to address in reports of Mendelian randomization studies[1 2]

Item No.	Section		Checklist item	Page No.	Relevant text from manuscript
1	**TITLE and ABSTRACT**		Indicate Mendelian randomization (MR) as the study's design in the title and/or the abstract if that is a main purpose of the study	1-2	"…To evaluate the causal relationship between oral microbiota and DSCs, we employed Genome-wide association study (GWAS) summary statistics for both oral microbiota and DSCs, in conjunction with bidirectional two-sample Mendelian randomization (MR) analysis…"
	**INTRODUCTION**				
2	**Background**		Explain the scientific background and rationale for the reported study. What is the exposure? Is a potential causal relationship between exposure and outcome plausible? Justify why MR is a helpful method to address the study question	2-3	"…Efforts to establish the association between the oral microbiota and all DSCs are critical for global DSC prevention and prognosis"
3	**Objectives**		State specific objectives clearly, including pre-specified causal hypotheses (if any). State that MR is a method that, under specific assumptions, intends to estimate causal effects	3	"Mendelian randomization (MR) is a widely conducted method that uses genetic variants to explore the relationship between exposures and outcomes…"
	**METHODS**				
4	**Study design and data sources**		Present key elements of the study design early in the article. Consider including a table listing sources of data for all phases of the study. For each data source contributing to the analysis, describe the following:	3-5	
		a)	Setting: Describe the study design and the underlying population, if possible. Describe the setting, locations, and relevant dates, including periods of recruitment, exposure, follow-up, and data collection, when available.	3	"In the context of this study, the two-sample MR method was employed to evaluate the possible causal link between oral microbiota and DSCs…"
		b)	Participants: Give the eligibility criteria, and the sources and methods of selection of participants. Report the sample size, and whether any power or sample size calculations were carried out prior to the main analysis	3	"…It comprises of 2017 samples from the tongue dorsum and 1915 saliva samples, and it covers a total of 10,098,668 loci…"
		c)	Describe measurement, quality control and selection of genetic variants	4	"These datasets were chosen based on strict selection criteria including…"
		d)	For each exposure, outcome, and other relevant variables, describe methods of assessment and diagnostic criteria for diseases	4-5	"For reverse analysis, the GWAS data of seven types of DSCs…"
		e)	Provide details of ethics committee approval and participant informed consent, if relevant	NA	NA

5	**Assumptions**		Explicitly state the three core IV assumptions for the main analysis (relevance, independence and exclusion restriction) as well assumptions for any additional or sensitivity analysis	5	"To ensure the rigor of a two-sample MR analysis, it must satisfy three key assumptions…"
6	**Statistical methods: main analysis**		Describe statistical methods and statistics used	5-6	
		a)	Describe how quantitative variables were handled in the analyses (i.e., scale, units, model)	5-6	"To ensure accuracy in inferring the causal relationship between oral microbiota and DSCs…"
		b)	Describe how genetic variants were handled in the analyses and, if applicable, how their weights were selected	5	"…SNPs were chosen with a MAF greater than"
		c)	Describe the MR estimator (e.g. two-stage least squares, Wald ratio) and related statistics. Detail the included covariates and, in case of two-sample MR, whether the same covariate set was used for adjustment in the two samples	5	"We employed five methods to evaluate the relationship between oral microbiota and gastrointestinal tumors…"
		d)	Explain how missing data were addressed	NA	NA
		e)	If applicable, indicate how multiple testing was addressed	5	"To control for multiple testing, we applied the False Discovery Rate (FDR) correction to the p-values obtained from the MR results for each tumor…"
7	**Assessment of assumptions**		Describe any methods or prior knowledge used to assess the assumptions or justify their validity	5-6	"…Additionally, for exposures involving multiple SNPs, heterogeneity tests, pleiotropy assessments, and leave-one-out analyses were conducted using the two-sample MR package"
8	**Sensitivity analyses and additional analyses**		Describe any sensitivity analyses or additional analyses performed (e.g. comparison of effect estimates from different approaches, independent replication, bias analytic techniques, validation of instruments, simulations)	6	"…we conducted a mediation analysis…"
9	**Software and pre-registration**			6	
		a)	Name statistical software and package(s), including version and settings used	6	"All statistical analyses were conducted using R software…"
		b)	State whether the study protocol and details were pre-registered (as well as when and where)	NA	NA
	**RESULTS**				
10	**Descriptive data**				

		a)	Report the numbers of individuals at each stage of included studies and reasons for exclusion. Consider use of a flow diagram	NA	NA
		b)	Report summary statistics for phenotypic exposure(s), outcome(s), and other relevant variables (e.g. means, SDs, proportions)		Supplementary Table 1-2
		c)	If the data sources include meta-analyses of previous studies, provide the assessments of heterogeneity across these studies	NA	NA
		d)	For two-sample MR:<br>i. Provide justification of the similarity of the genetic variant-exposure associations between the exposure and outcome samples<br>ii. Provide information on the number of individuals who overlap between the exposure and outcome studies	NA	NA
11	**Main results**			6-11	
		a)	Report the associations between genetic variant and exposure, and between genetic variant and outcome, preferably on an interpretable scale	6-11	"Bidirectional MR analysis was conducted to examine the causal relationships between oral microbiota and seven distinct types of DSCs…"
		b)	Report MR estimates of the relationship between exposure and outcome, and the measures of uncertainty from the MR analysis, on an interpretable scale, such as odds ratio or relative risk per SD difference	6-11	"…We discovered that the genetically predicted relative abundance of genus *Prevotella*…"
		c)	If relevant, consider translating estimates of relative risk into absolute risk for a meaningful time period	NA	NA
		d)	Consider plots to visualize results (e.g. forest plot, scatterplot of associations between genetic variants and outcome versus between genetic variants and exposure)		Figure2-3
12	**Assessment of assumptions**				
		a)	Report the assessment of the validity of the assumptions		Supplementary Table 5
		b)	Report any additional statistics (e.g., assessments of heterogeneity across genetic variants, such as I^2, Q statistic or E-value)		Supplementary Table 6
13	**Sensitivity analyses and additional analyses**			11	
		a)	Report any sensitivity analyses to assess the robustness of the main results to violations of the assumptions	11	"All pleiotropy tests yielded non-significant results (p > 0.05)…"

		b)	Report results from other sensitivity analyses or additional analyses	11	"…we applied both the IVW method and MR-Egger regression…"
		c)	Report any assessment of direction of causal relationship (e.g., bidirectional MR)	NA	NA
		d)	When relevant, report and compare with estimates from non-MR analyses	NA	NA
		e)	Consider additional plots to visualize results (e.g., leave-one-out analyses)		Supplementary Table 5-6
	**DISCUSSION**				
14	**Key results**		Summarize key results with reference to study objectives	11-17	"To the best of our knowledge, this research is one of the pioneering attempts to systematically assess the causal relationships between the oral microbiota and DSCs through a bidirectional two-sample MR study…"
15	**Limitations**		Discuss limitations of the study, taking into account the validity of the IV assumptions, other sources of potential bias, and imprecision. Discuss both direction and magnitude of any potential bias and any efforts to address them	16	"The current study also has several limitations…"
16	**Interpretation**				
		a)	Meaning: Give a cautious overall interpretation of results in the context of their limitations and in comparison with other studies	15	"However, it is noteworthy that the absence of several experimentally validated oral microbes…"
		b)	Mechanism: Discuss underlying biological mechanisms that could drive a potential causal relationship between the investigated exposure and the outcome, and whether the gene-environment equivalence assumption is reasonable. Use causal language carefully, clarifying that IV estimates may provide causal effects only under certain assumptions	15	"Indeed, a single type of bacterium can play contrasting roles in cancer development…"
		c)	Clinical relevance: Discuss whether the results have clinical or public policy relevance, and to what extent they inform effect sizes of possible interventions	17	"For clinicians, this implies that modulating the oral microbiota could be a promising strategy…"
17	**Generalizability**		Discuss the generalizability of the study results (a) to other populations, (b) across other exposure periods/timings, and (c) across other levels of exposure	16	"To enhance the generalizability of these results, future MR studies should aim to include a more diverse cohort, encompassing both Asian and non-Asian populations…"
	**OTHER INFORMATION**				
18	**Funding**		Describe sources of funding and the role of funders in the present study and, if applicable, sources of funding for the databases and original study or studies on which the present study is based	18	"Not applicable"

19	**Data and data sharing**	Provide the data used to perform all analyses or report where and how the data can be accessed, and reference these sources in the article. Provide the statistical code needed to reproduce the results in the article, or report whether the code is publicly accessible and if so, where	17	“All data generated or analysed during this study are included in this published article and its supplementary information files”
20	**Conflicts of Interest**	All authors should declare all potential conflicts of interest	18	“The authors declare that the research…”

This checklist is copyrighted by the Equator Network under the Creative Commons Attribution 3.0 Unported (CC BY 3.0) license.

1. Skrivankova VW, Richmond RC, Woolf BAR, Yarmolinsky J, Davies NM, Swanson SA, et al. Strengthening the Reporting of Observational Studies in Epidemiology using Mendelian Randomization (STROBE-MR) Statement. JAMA. 2021;under review.

2. Skrivankova VW, Richmond RC, Woolf BAR, Davies NM, Swanson SA, VanderWeele TJ, et al. Strengthening the Reporting of Observational Studies in Epidemiology using Mendelian Randomisation (STROBE-MR): Explanation and Elaboration. BMJ. 2021;375:n2233.
